# Comparison of four different assays to evaluate cellular-mediated immunity against cytomegalovirus in solid organ transplantation

**DOI:** 10.3389/fimmu.2025.1567253

**Published:** 2025-05-16

**Authors:** Angela Casas-Parra, Hendrik Veltman, Alba Romero-Caballero, Rosana Gelpi-Remiro, Marc Boigues-Pons, Imán Allalou, Ian Linares-Pardo, Anna Vila-Santandreu, Eva Martínez-Cáceres, María Iglesias-Escudero

**Affiliations:** ^1^ Division of Nephrology, Germans Trias i Pujol University Hospital, Badalona, Spain; ^2^ Germans Trias I Pujol Research Institute (IGTP), Badalona, Spain; ^3^ Immunophatology Group, Germans Trias I Pujol Research Institute (IGTP), Badalona, Spain; ^4^ Division of Infectious Diseases, Germans Trias i Pujol Hospital, Badalona, Spain; ^5^ Fundacio´ Lluita contra les Infeccions, Germans Trias i Pujol University Hospital Badalona, Badalona, Spain; ^6^ Division of Immunology, LCMN, Germans Trias I Pujol Hospital, Badalona, Spain; ^7^ Department of Cell Biology, Physiology and Immunology, Autonomous University of Barcelona (UAB), Badalona, Spain

**Keywords:** cytomegalovirus, solid organ transplantation, specific cellular immune response, immune monitoring, T cell response

## Abstract

CMV infection is the most prevalent opportunistic infection following solid organ transplantation (SOT), significantly affecting both graft and patient survival. Effective control of viral replication is crucial to prevent CMV infection from progressing to end-organ disease. Despite its high prevalence, options for preventing CMV infection and end-organ disease are limited to a few antiviral drugs, which have severe side effects and may lead to resistance. In this context, measuring CMV-specific cell-mediated immunity (CMI) has proven to be a valuable tool, with high negative predictive value (NPV) for the absence of CMV viremia in patients with positive tests. This study aimed to evaluate the sensitivity and specificity of various cellular immune response assays and assess the feasibility of incorporating them into routine clinical practice for kidney transplant recipients (KTR). Conducted at the Hospital Universitari Germans Trias i Pujol (HGTP), the study analyzed 56 samples from KTR and 10 healthy controls (HC). Patients and controls were classified based on their pre-transplant serostatus, and CMI was measured using QuantiFERON-CMV^®^ ELISA, T cell proliferation assay (TCPA), activation-induced marker (AIM) assay, and an in-house ELISA. The AIM assay demonstrated that CD69 is a reliable activation marker for flow cytometry-based assays, as it consistently increased following polyclonal stimulation. Notably, among the total patient cohort with CD4 T cell reactivity, the CM subpopulation exhibited the most significant increase (p < 0.001). Comparative analysis revealed that both ELISAs had high sensitivity and specificity compared to other techniques. The consistency test results showed perfect and almost perfect agreement between the AIM (cut-off 0.2) and the QuantiFERON-CMV^®^ ELISA and in-house ELISA, respectively. The study also explored the feasibility of incorporating these tests into daily clinical practice, proposing an algorithm based on test results and cost-effectiveness. This algorithm involves testing patients using the QuantiFERON-CMV^®^ assay, followed by AIM testing in cases of indeterminate results or HLA mismatches. Incorporating these assays would help identify patients at the lowest risk of CMV infection after prophylaxis, enabling more selective and personalized prophylactic strategies.

## 1 Introduction

Advances in immunosuppressive therapies have significantly reduced the incidence of acute rejection in organ transplantation. However, maintaining the delicate balance between preventing organ rejection and avoiding complications from immunosuppressive therapies, such as increased susceptibility to infections, remains a common challenge in organ transplantation (1).

CMV infection is the most prevalent opportunistic infection following SOT, negatively impacting both graft and patient survival (2). Therefore, effective management of CMV infection is crucial for improving transplant outcomes (3). CMV is a double-stranded DNA virus from the Herpesviridae family. Its prevalence in the general population approaches 75%, with seroprevalence ranging from 50% to 96%, depending on the geographic region (4, 5). Primary infection typically occurs in childhood, and the virus remains in a latent form in the host indefinitely. In the general population, CMV infection is often asymptomatic or minimally symptomatic; however, in KTR, it can manifest in a wide range of clinical forms and organ involvement, which are associated with significant morbidity and mortality.

After transplantation, CMV infection may arise either from reactivation of a pre-existing latent infection in the recipient’s organ or from a primary infection caused by the organ donor. Approximately 75% of transplant recipients exhibit evidence of active CMV infection within the first year post-transplantation (6). The risk of CMV infection is influenced by the recipient’s serostatus and the immunosuppression regimen used after kidney transplantation, with the highest risk occurring during the first year post-transplantation.

CMV infection triggers a range of immune responses, involving both innate and adaptive components. The immune system’s ability to recognize and respond to CMV is critical in preventing the virus from causing severe disease. Among the various immune responses, CMI is particularly important in the context of CMV infection. CMI involves the activation of immune cells, particularly memory/effector T cells, which play a central role in combating viral infections (7). Effective control of viral replication is essential to prevent progression to end-organ disease, and a lack of immune control by CMV-specific T cells may indicate individuals at increased risk of CMV disease after transplantation.

Incorporating the monitoring of cellular immunity against CMV (CMI anti-CMV) is useful in adjusting the duration of prophylaxis for patients at high risk of CMV disease. This approach could serve as a guideline to reduce the duration of antiviral prophylaxis, thereby minimizing the risk of toxicity, with minimal impact on the incidence of CMV-related disease (8–12). Several observational studies have been published using different techniques to monitor T cell immunity against CMV in the pre and post-transplant context (12–14). However, the ideal method in terms of accuracy, ease of Implementation, assay standardization and short turnaround time remains under investigation (14, 15).

The objective of this report is to assess the feasibility and practicality of integrating specific cellular immune response monitoring into daily clinical practice for KTR. This includes considerations of sensitivity, specificity, cost-effectiveness, and ease of implementation in routine clinical care.

## 2 Materials and methods

### 2.1 Study design

The study was conducted on 56 samples obtained from intermediate- and high-risk KTR at the HGTP since 2023. The study was approved by the HGTP Ethics Committee (CEIC). All patients and healthy controls (HC) provided informed consent and agreed to participate in the study. Cellular immunity samples were collected within the first year after transplantation. All patients were placed on a standard immunosuppression regimen consisting of triple therapy with steroids, tacrolimus, and mycophenolate. The sample collection for patients that received induction therapy with thymoglobulin, started from the third month post-transplant. On the other hand, for the patients that received induction with basiliximab, sample collection started from the first month post-transplant. Sample collection was conducted in a fasting state, prior to the morning dose of tacrolimus.

Patients were classified based on their pre-transplant serostatus (CMV-specific IgG) and immunosuppression into the following groups: high risk (D+/R-), intermediate risk (R+ with thymoglobulin), and intermediate risk (R+). A group of 10 HC was tested for cellular immunity against cytomegalovirus (CMV) and all individuals were found to be negative for CMV-specific IgG antibodies.

### 2.2 T cell proliferation assay

Human PBMCs were isolated from peripheral blood of HC and KTR using Ficoll density gradient centrifugation (Lymphoprep™; Serumwerk Bernburg AG, Germany). Fresh PBMCs were labelled with VPD-450 (BD Biosciences, US). A total of 1 × 10^7^ cells/ml were stained with 1 μM VPD-450 for 15 min at 37°C in the dark (16). After washing, PBMCs were resuspended in 1 ml supplemented RPMI (RPMI, 2% L-Glutamine, 1% non-essential amino acids, 0.5% Penicillin/Streptomycin, 10% autologous serum). A total of 1.5 × 10^5^ PBMCs were seeded in 96-well plates (12 wells, 200 μl/well) for 7 days at 37°C with a CMV peptide pool (5 μg/ml) (California Pep Mix, JPT). Twelve wells without antigen served as a negative control, and six wells with PHA (Sigma-Aldrich, 1 µg/mL) were used as a positive control to assess PBMC integrity.

In proliferating T cells, the VPD-450 dye is equally distributed between daughter cells, resulting in multiple peaks, each displaying half the fluorescence intensity with each cell division. This enables tracking of cell division through successive generations. Results are expressed as the percentage of proliferating CD3+ VPD-450dim cells.

To evaluate antigen-specific CD4+ T cell responses (16), the mean plus 3 standard deviations (SD) of the unstimulated wells was used as the cut-off for a result to be considered positive. To evaluate the accuracy of the established cut-off, low-positive samples were considered positive.

### 2.3 Activation induced marker assay

Three hundred microliters of heparinized whole blood were stimulated with a CMV peptide pool (5 μg/ml). Non-stimulated and PHA-stimulated whole blood (1 µg/mL) served as negative and positive controls, respectively. The samples were incubated for 17 hours at 37°C, 5% CO_2_, and 95% humidity. Stimulated blood was stained to evaluate the expression of the following markers: CD3, CD4, CD8, CCR7, CD45RA, CD69, CD25, OX40, and CD40L, and incubated for 20 minutes in the dark at room temperature (RT). After incubation, the samples were lysed, washed, and acquired using a Fortessa^®^ flow cytometer (BD Biosciences).

In our testing, a result was considered positive when the percentage of T cells expressing activation markers (CD69, CD25, OX40, and CD40L) was at least double (100%) the value observed in the negative control. A sample was classified as negative if the percentage of activated marker expression was less than 20% of the expression observed in the negative control. To evaluate the accuracy of the cut-off, two different thresholds were assessed in the study: a cut-off of 0.2 (when marker expression was 20% higher than the negative control) and a cut-off of 2 (when marker expression was double the negative control). The results were calculated based on the percentage of CD3+ cells.

### 2.4 Monoclonal antibodies and flow cytometry analysis

To evaluate the AIMs, the following monoclonal antibodies were used: Anti-CD45RA Fluorescein isothiocyanate (FITC) (Clone L48, Isotype Mouse BALB/c IgG1, κ, Status CE_IVD, BD Biosciences), Anti-OX40 Phycoerytrin (PE) Clone 134–1 Isotype IgG1 cytognos CYT-134PE Status CE_IVD, Anti-CD4 Peridinin-Chlorophyll-Protein 5.5 (PerCPCy5.5) Clone RPA- Isotype Mouse IgG1, *κ* T4 Pharmingen Status RUO, Anti-CD25 (PE)-cyanin 7 (Cy7) Clone 2A3 Isotype Mouse BALB/c IgG1, *κ* BD Status CE_IVD, Anti-CCR7 PE-CF594 Clone 150503 Isotype Mouse IgG2*α* BD Status RUO, Anti-CD40L Brilliant Violet 605 (BV421) Clone TRAP1 Isotype Mouse BALB/c IgG1, *κ* BD Status RUO, Anti-CD8 Allophycocyanin APC-H7 Clone SK1 Isotype Mouse BALB/c IgG1, *κ* BD Status RUO, Anti-CD3 Violet 500 (V500) Clone UCHT1 Isotype Mouse BALB/c IgG1, *κ* BD Status RUO, Anti-CD69 Brilliant Violet 605 (BV605) Clone FN50 Isotype Mouse IgG1, *κ* Biolegend Status RUO. Fluorescence Minus One controls were used to assess the background signal for the CD69, CD25, OX40 and CD40 Ligand (L) expression markers. Samples were stained and incubated for 20 minutes in the dark at room temperature (RT). After incubation, the samples were lysed, washed, and acquired using a Fortessa^®^ flow cytometer (BD Biosciences).

To evaluate T cell proliferation, PBMCs were stained with the following monoclonal antibodies: Anti-CD3 APC-H7 Clone SK7, Mouse BALB/c IgG1, *κ*, BD CE_IVD. CD4 FITC Clone L48, Isotype Mouse BALB/c IgG1, κ, Status CE_IVD, BD Biosciences, Phycoerythrin (PE) Clone 134–1 Isotype IgG1 cytognos CYT-134PE Status CE_IVD. Samples were stained for 30 min at 4°C in the dark. PBMCs collected after culture were incubated during 20 min, washed with Phosphate Buffer Saline (PBS) and acquired in a Fortessa^®^ flow cytometer. Samples were acquired with FACS Canto II (BD Bioscience) and flow cytometry data was analyzed using Diva Software.

### 2.5 Analysis of cell viability

To assess the frequencies of cell death in *in vitro* cell culture conditions, collected cells from the plates were incubated for 10 minutes with 7-aminoactinomycin D (7AAD) (7-AAD BD Pharmingen RUO) before acquisition. The dead cells can then be identified and removed from the final analysis by gating on the unstained population (live cells).

### 2.6 IFN-*γ* production measured by ELISA

Three hundred microliters of heparinized whole blood were stimulated with CMV peptides (5 µg/mL). Phytohemagglutinin (PHA) (1 µg/mL) served as a positive control. The samples were incubated for 17 hours at 37°C and 5% CO_2_. Subsequently, the supernatant was removed, and the level of IFN-γ was measured via ELISA according to the manufacturer’s instructions (Legend Max Human IFN-γ ELISA Kit, Cat No. 430107, BioLegend). A four-parameter logistic curve was used to determine the IFN-γ concentrations.

A result was considered negative if the difference between the CMV antigen tube and the negative control was less than 0.2 IU/mL. A result was considered positive if the difference was greater than 0.2 IU/mL. The minimum IFN-γ concentration in the mitogen control, based on our study population, was set at >0.7 IU/mL to validate the assay.

### 2.7 IFN-*γ* production measured by Quantiferon-CMV ELISA

The QuantiFERON-CMV (QF-CMV^®^) assay was performed according to the manufacturer’s instructions (Qiagen). One milliliter of heparinized blood was collected into each of the QF-CMV blood collection tubes: the null control tube, the CMV antigen tube, and the mitogen tube (positive control). The tubes were incubated at 37°C within 16 hours of blood collection. After a 17-hour incubation period, supernatants were collected and analyzed for IFN-γ concentration (IU/mL) using standard ELISA.

According to the manufacturer’s instructions, a result for the CMV antigen tube was considered “reactive” when the CMV antigen response, minus the negative control, was greater than 0.2 IU/mL of IFN-γ. A result was considered “indeterminate” when the IFN-γ concentration in the CMV antigen tube minus the negative control was less than 0.2 IU/mL, and the IFN-γ concentration in the mitogen tube (after subtracting the negative control) was less than 0.5 IU/mL. All participants enrolled in the study had HLA class I alleles capable of binding CMV peptides.

### 2.8 Peptides pools

ELISA CMV, TCPA and AIM assay were stimulated with PepTivator^®^ CMV pp65 (5 μg/ml) (Miltenyi Biotec). Those peptide pools of 15–aa length with 11–aa overlap that have been developed for the *in vitro* stimulation of antigen-specific CD4+ and CD8+ T cells. The Quantiferon CMV assay includes CMV peptides to target CD8+ T lymphocytes, including class I HLA haplotypes A1, A2, A3, A11, A23, A24, A26, B7, B8, B27, B35, B40, B41, B44, B51, B52, B57, B58, B60, and Cw6 (A30, B13), which cover >98% of the human population.

### 2.9 Statistical analysis

To test if the variables followed a Gaussian distribution we performed Kolmogorov Smirnoff test. For those nonparametric unpaired variables, Mann-Whitney U test was used to compare two groups and Kruskal-Wallis (not matching) or Friedman (repeated measures) test were used to compared more than two groups. To check parametric unpaired variables Student´s t test was used to compare two groups and more than two groups were compared using the parametric analysis of variance (ANOVA) as appropriate. Differences between two paired groups were assessed using the Student´s t-test for paired data or the Wilcoxon signed-rank test when data were or not normally distributed, respectively. Multiple comparisons were assessed using Dunn or Tukey´s tests. Cohen’s kappa was calculated (AI) to assess the level of agreement or consistency between two measurement methods. Statistical analyses were performed using GraphPad software version 8.4.3 (GraphPad Inc. San Diego, CA).

## 3 Results

### 3.1 Analysis of the TCPA

**Figure 1:**
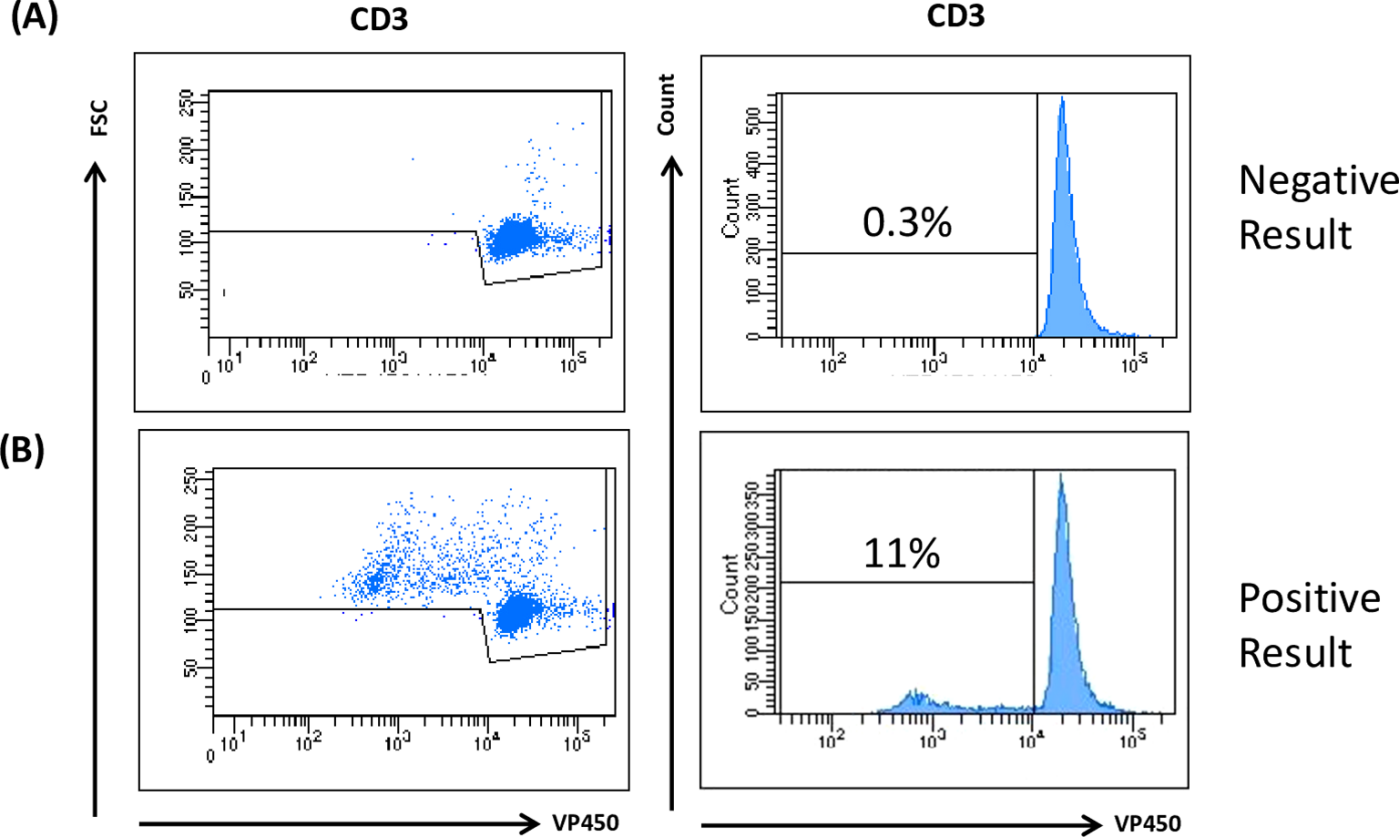
TCPA analysis. Human PBMCs were isolated from peripheral blood obtained from both HC and KTR by Ficoll density gradient centrifugation. Cells were incubated upon stimulation for 7 days with 5 µg/mL CMV and PHA. VPD450 marker was used to evaluate T cell proliferation. A positive test result indicates that the patient exhibits CMV immunity due to the reaction upon the virus antigen. Cuff off value: 3SD of the Negative Control. The figure is representative of a negative **(A)** and positive **(B)** result.

TCPAs are used to measure the ability of T cells to proliferate in response to antigens, mitogens, or cytokines. This test is based on the stimulation of lymphocytes isolated from blood samples to assess their clonal activation and proliferation upon CMV stimulation (8). **Figure 1** shows representative results for a negative (**a**) and a positive (**b**) outcome. TCPA allows analysis of CD3+ cell proliferation as well as CD4+ and CD8+ responses, with a minimum assay time of 5–7 days. Its implementation requires experienced personnel and flow cytometry equipment.

Patients and controls were classified according to their pre-transplant serostatus (CMV-specific IgG): intermediate risk (R+, n=40) and high risk (D+/R-), n=12 (**Supplementary Table 1**). A positive result was obtained in 54% (n=28) of the tested patients, while 46% (n=24) were negative. All healthy donors (n=10) who were CMV IgG negative, tested negative in the TCPA (**Supplementary Table 2**).

### 3.2 AIM flow cytometry-based assay

**Figure 2:**
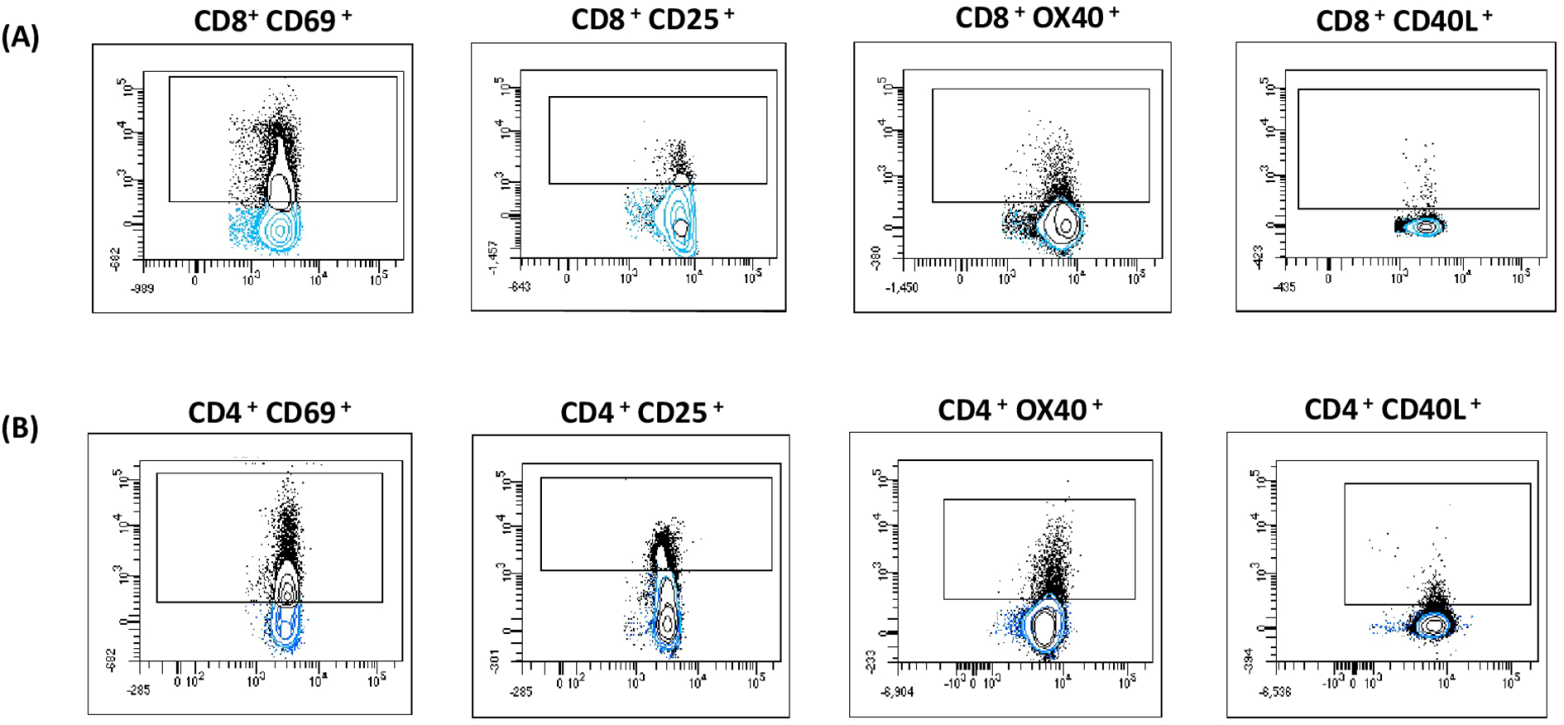
AIM assay. After incubation the cells were stained with monoclonal antibodies and the expression of activation-induced markers was analyzed by flow cytometry. The following activation markers: OX40+ CD40L+, CD69+ CD25 were used to evaluate CD8+ **(A)** and CD4+ **(B)** CMV-Specific reactivity. Gated cells (black dots) are considered positive. Populations were expressed as a percentage of cells expressing the marker in relation to the total CD3, CD4 or CD8 T cells.

Activation markers can be expressed on both central memory T cells (TCM) and effector memory T cells (TEM) upon activation. We aimed to determine whether information could be obtained from these distinct cell types. The expression of activation-induced markers was analyzed by flow cytometry, as described in **Figures 2A, B** and **Supplementary Figure 1**. Patients and controls were classified according to their pre-transplant serostatus (CMV-specific IgG) as follows: intermediate risk (R+, n=34) and high risk (D+/R-, n=4). When the cut-off was set at 20% above the negative control (cut-off 0.2), a positive result was obtained in 63% (n=24) of the evaluated patients, while 37% (n=14) were negative. Among the 10 healthy donors (tested as IgG CMV negative), 100% (n=10) were AIM negative. (**Supplementary Table 3**).

Conversely, when the cut-off was set at 100% above the negative control (cut-off 2), a positive result was obtained in 24% (n=9) of the patients under study, while 76% (n=29) were negative. All 10 healthy donors tested as IgG CMV negative were AIM negative. (**Supplementary Table 4**).

The AIM assay offers functional and quantitative data on CMV-specific CD4+ and CD8+ T cells. It provides results within 24 hours and can be easily implemented in labs with flow cytometry access.

### 3.3 CD69 as an activation marker of T cells in flow cytometry-based assays

**Figure 3:**
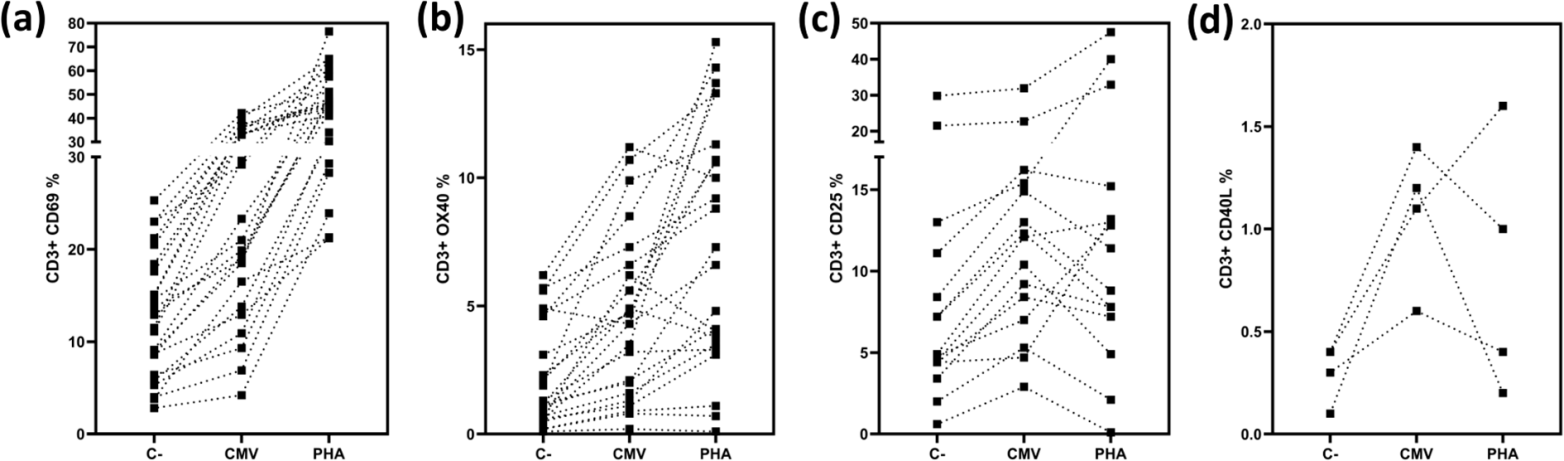
Expression of activation markers in CD3 T cells. Depicts the percentages of positive and borderline CD3 T-cells expressing CD69 **(a)** depicts the percentages of positive and borderline CD3 T-cells expressing CD25 **(b)**, depicts the percentages of positive and borderline CD3 T-cells expressing OX40 **(c)**, depicts the percentages of positive and borderline CD3 T-cells expressing CD40L **(d)** Negative controls, CMV stimulated samples and positive controls are shown.

As previously described, the markers OX40, CD40L, CD69, and CD25 were used to evaluate T cell reactivity. The samples with positive and borderline results for each marker are depicted in **Figure 3**.

When evaluating CD3+ T cells expressing CD69, we found that 21% of the samples were positive (n=9), and 34% were borderline (n=15) (n=38) (**Figure 3**). Analyzing CD3+ T cells expressing CD25, we found that 13% of the samples were positive (n=6), and 19% were borderline (n=9) (n=46) (**Figure 3**). For the marker OX40, 28% of the samples were positive (n=13), and 21% were borderline (n=10) (n=46) (**Figure 3**). Finally, when analyzing CD3+ T cells expressing CD40L (**Figure 3**), 18% of the samples were positive (n=4) (n=22).

Since CD69 was the only marker in our assay that consistently increased after polyclonal stimulation (**Figure 3**), it became a crucial element for accurate interpretation. Therefore, we considered the evaluation of CD69 alone sufficient to assess T cell reactivity against CMV.

Based on these results, we determined the positivity or negativity of the samples, considering as valid only those assays where the positive control exhibited expression at least 20% higher than that observed in the CMV-stimulated wells. Consequently, the AIM results presented in the previous section were exclusively based on the evaluation of CD69 expression.

### 3.4 Central memory is the most informative subpopulation among the CD4 T cells in the flow cytometry-based assay

The Activation-Induced Marker (AIM) assay not only enables the assessment of the activation status of CD4+ and CD8+ T cells, but also allows for the categorization of T lymphocytes into functionally distinct populations using a combination of markers: naïve (CCR7+ CD45RA+), central memory (CM) (CCR7− CD45RA−, CM), effector memory (EM) (CCR7− CD45RA−, EM), and terminally differentiated effector memory (CCR7− CD45RA+, EMRA) T cells (**Supplementary Figure 1**).

**Figure 4:**
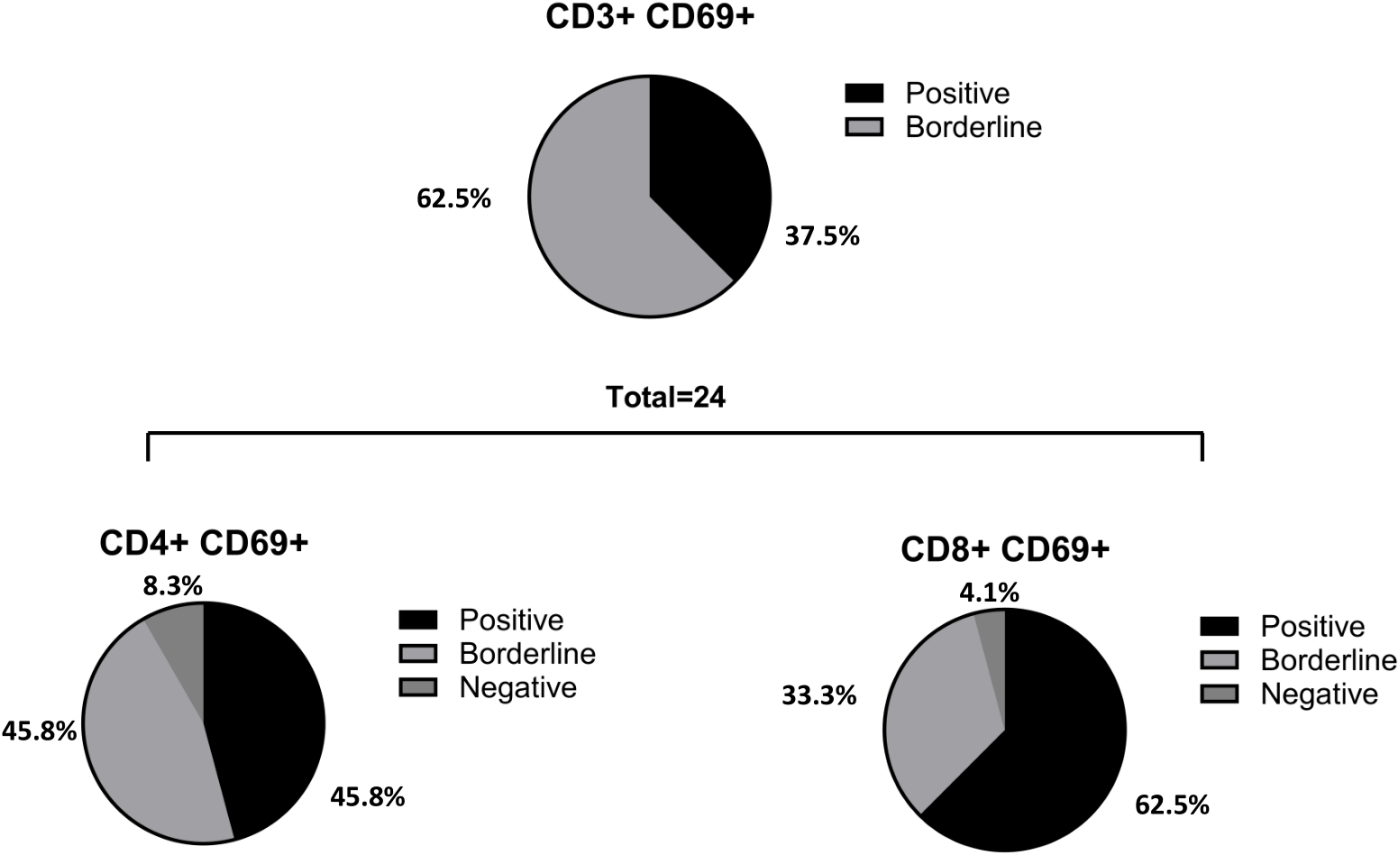
Percentage of positive, borderline and negative patients in CD3, CD4 and CD8 T cells. From the total number of patients analyzed with the AIM (n= 38), 9 (37.5%) were CD3^+^ CD69^+^ positive and 15 (62.5%) borderline (negatives excluded in percentage calculation). When CD4 and CD8 populations were evaluated, it was observed that 11 patients (45.8%) were CD4^+^ CD69^+^ positive, 11 patients (45.8%) were borderline and 2 (8.3%) were negative (n= 24); 15 patients (62.5%) were CD8^+^ CD69^+^ positive, 8 (33.3%) borderline and 1 (4.1%) patient was negative (n= 24). In one of the patients, it was detected a CD4 positive result that did not show CD8 reactivity.

As outlined in previous results, of the 38 patients analyzed using the AIM assay, 9 (37.5%) were CD3+ CD69+ positive, and 15 (62.5%) exhibited borderline results. When CD4+ and CD8+ T cell populations were assessed, we found that 11 patients (45.8%) were CD4+ CD69+ positive, 11 patients (45.8%) were borderline, and 2 (8.3%) were negative (n=24). Similarly, 15 patients (62.5%) were CD8+ CD69+ positive, 8 (33.3%) were borderline, and 1 (4.1%) was negative (n=24) (**Figure 4**).

In summary, two patients with CD8+ reactivity did not show CD4+ reactivity, whereas one patient displayed CD4+ reactivity and was CD8+ negative. In these three cases, the overall performance of the AIM assay was classified as borderline.

**Figure 5:**
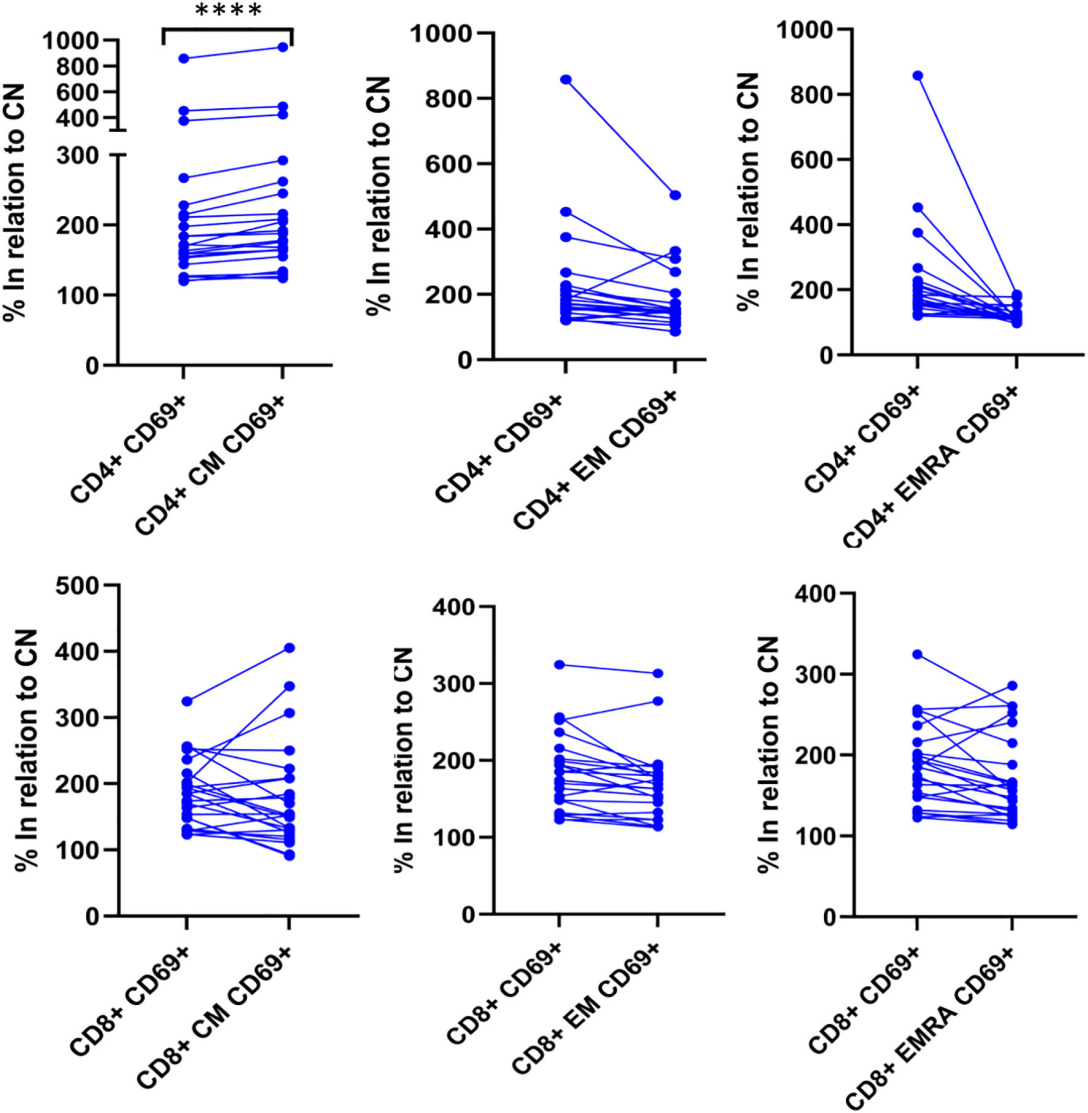
CM is the most informative subpopulation among the CD4 T cells. The degree of T-lymphocyte activation and differentiation was analyzed by flow cytometry. Upon activation, naïve T-cells further differentiate into their memory subpopulations, including CM, EM and lastly, EMRA T-cells. In positive samples, the expression of CD69 on CM CD4+ cells was the highest among the CD4 subpopulations. The expression of CD69 is significant higher (p<0.0001) in CD4+ CM compared to total CD4+ when the results are normalized to the negative control. **** p<0.0001 Differences between groups were assessed by Wilcoxon signed-rank test.

We further examined whether the analysis of different T cell subpopulations could provide additional information. As expected, naïve CD4+ and CD8+ T cells showed no CMV reactivity or polyclonal activation. Additionally, the number of events in the EMRA subsets was low and likely not representative. However, the CM and EM populations provided the most meaningful information. When analyzing the total cohort of patients with CD4+ reactivity, we observed that the CM population showed the greatest increase (p<0.001) compared to the overall CD4+ T cell population (**Figure 5**). In contrast, when CD8+ cell reactivity was assessed, analyzing the subpopulations did not yield additional insights compared to the overall reactive CD8+ population (**Figure 5**). To reduce variability, the results were normalized to the control negative (CN) value for each sample.

### 3.5 IFN-*γ* production measured by ELISA

In brief, heparinized whole blood samples were stimulated with CMV peptides, incubated for 17 hours, and the concentration of IFN-γ was measured via ELISA according to the manufacturer’s instructions. Patients and controls were classified based on their pre-transplant serostatus (CMV-specific IgG) as follows: intermediate risk (R+, n=18) and high risk (D+/R-, n=7). A positive result was obtained in 80% (n=20) of the tested patients, while 20% (n=5) were negative. All healthy donors (n=10), who were CMV IgG negative, also tested negative for IFN-γ production by ELISA (**Supplementary Table 5**).

### 3.6 IFN-*γ* production measured by QUANTIFERON CMV ELISA

The QuantiFERON-CMV assay is an ELISA that measures IFN-γ release by CD8+ T cells. It is commercially available in certain regions (CE-marked in Europe) and has been clinically evaluated in kidney transplant patients (KTP) at high risk for CMV infection, demonstrating its predictive value for CMV disease. Patients and controls were classified according to their pre-transplant serostatus (CMV-specific IgG) as follows: intermediate risk (R+, n=45) and high risk (D+/R-, n=11). A positive result was observed in 80% (n=36) of the tested patients, while 20% (n=9) were negative. All healthy donors (n=10), who were CMV IgG negative, tested negative in the QuantiFERON-CMV assay (**Supplementary Table 6**). As an ELISA, the QuantiFERON-CMV assay is relatively simple to perform, can be automated, and provides quick results.

### 3.7 Comparison of the TCPA, AIM, IFN-γ production measured ELISA and by Quantiferon CMV ELISA

From the findings, we calculated analytical sensitivity, specificity, and predictive values for the four tests as follows: TCPA showed a sensitivity of 54%, specificity 100% NPV 29%, positive predictive value (PPV) 100%. AIM (cut-off 0.2): Sensitivity 63%, Specificity 100%, NPV 41%, PPV 96%. AIM (cut-off 2): Sensitivity 24%, Specificity 100%, NPV 42%, PPV 100%. IFN-γ ELISA (cut-off 0.2): Sensitivity 80%, Specificity 100%, NPV 67%, PPV 100%. QF CMV ELISA (cut-off 0.2): Sensitivity 92%, Specificity 100%, NPV 53%, PPV 100% (**Table 1**).

**Table 1:** Sensitivity, specificity and predictive values in the four assays tested.

Technique	Sensitivity (%)	Specificity (%)	PPV (%)	NPV (%)
T cell proliferation assay (n = 52)	54	100	100	29
AIM (n=38) cut off 0.2	63	100	96	41
AIM (n=38) cut off 2	24	100	100	42
In-house ELISA (n=31)	80	100	100	67
Quantiferon-CMV (n=38)	92	100	100	53

AIM, Activation Induced Markers; PPV, positive predictive value; NPV, negative predictive value.

### 3.8 Consistency analysis of TCPA, AIM, ELISA and QF ELISA

**Figure 6:**
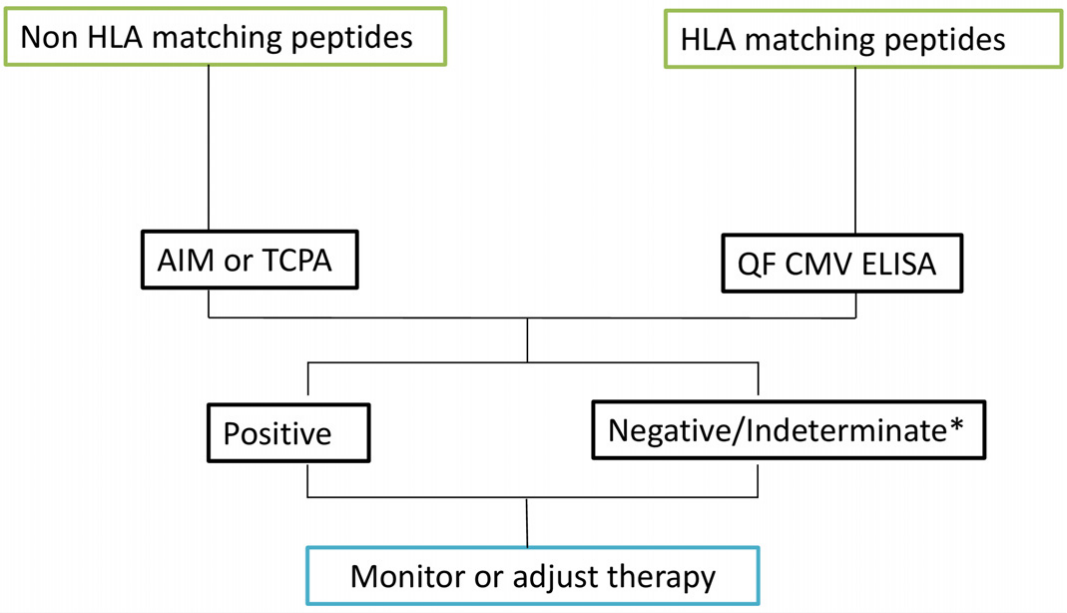
Algorithm based on results. Upon reviewing the HLA-matched peptides, select the most appropriate technique for further analysis. For HLA-matched peptides, proceed with the QF CMV ELISA test. For peptides that do not match the HLA, utilize either the AIM assay or the TCPA. Based on the results obtained from these assays, monitor the patient’s condition and adjust the therapeutic approach accordingly. * Indeterminate results are typically due to a low polyclonal response. In the case of indeterminate results, a new sample should be requested.

In addition to sensitivity, specificity, and predictive values, the performance of diagnostic tests or agreement between raters was assessed using consistency measures and the kappa (κ) index, which evaluates agreement between tests. The consistency analysis revealed the following results: AIM (cut-off 0.2) vs QF ELISA: κ = 1 (perfect agreement). AIM (cut-off 2) vs QF ELISA: κ = 0.297 (fair agreement, n=37). AIM (cut-off 0.2) vs TCPA: κ = 0.613 (substantial agreement, n=25). AIM (cut-off 2) vs TCPA: κ = 0.719 (substantial agreement, n=25). AIM (cut-off 0.2) vs ELISA: κ = 0.833 (almost perfect agreement). AIM (cut-off 2) vs ELISA: κ = 0.385 (fair agreement, n=12). TCPA vs ELISA: κ = 0.419 (moderate agreement, n=20). TCPA vs QF ELISA: κ = 0.545 (moderate agreement, n=37). ELISA vs QF ELISA: κ = 0.67 (substantial agreement, n=21) (**Supplementary Material 1**). As a result of the findings, considerations of sensitivity, specificity, cost-effectiveness and ease of implementation in routine clinical care we have implemented an algorithm to evaluate CMV T Cell specific responses (**Figure 6**).

## 4 Discussion

CMV infection is the most prevalent opportunistic infection after SOT, significantly impacting both graft survival and patient outcomes. The risk of CMV infection in the SOT context depends on several factors, including the CMV IgG status of both the donor and recipient, the immunosuppressive regimen used, and is highest during the first year post-transplantation (17). Therefore, preventing CMV infection is critical for improving transplant outcomes. Despite its high prevalence, the options for preventing CMV infection and end-organ disease remain limited, with only a few antiviral treatments available. These antivirals often come with severe side effects and may lead to drug resistance. Effective control of viral replication is crucial to prevent CMV progression to end-organ disease.

In this context, measuring CMV-specific CMI has shown a high NPV for the absence of CMV viremia in patients with a positive test result. Recent clinical trials have also demonstrated the utility of CMI monitoring in predicting CMV infections based on the patient’s immune status (18). This approach not only reduces the likelihood of side effects and antiviral resistance by allowing for earlier discontinuation of medication but also enables more personalized treatment strategies tailored to each patient’s individual immune response (18).

Despite the technical challenges in studying Ag-specific T cells, due to their low frequency in peripheral blood (19), various specialized assays have been developed and optimized for their quantification and characterization.

These assays typically rely on functional immune responses to specific antigens, such as the induction of T cell proliferation (19), cytokine production detected by intracellular cytokine staining (ICS), enzyme-linked immunosorbent assays (ELISA), enzyme-linked immune-spots (ELISpot) (20, 21), induction of activation markers analyzed by flow cytometry (22, 23), and MHC class II tetramer staining. The choice of assay depends on factors such as sample size, the need for high throughput, and the specific information required (24).

The advantages and limitations of each of these techniques have been extensively reviewed in the literature (8, 16, 17, 25). In this report, we present a comparative analysis of four distinct assays used to assess CMV-specific T cell responses: AIM assays analyzed by flow cytometry, ELISA, QF CMV ELISA, and TCPA.

Flow cytometry-based assays, such as the AIM and ICS assays, allow for the identification of both adaptive and innate immune cells within a single sample by using specific lineage markers. The use of AIM assays during the peak of the COVID-19 pandemic was instrumental in enabling various research groups to quickly assess robust T cell memory development following SARS-CoV-2 infection (25). These assays also facilitated the tracking of the kinetics of the T-cell response over time in large study cohorts, revealing that T cell memory persisted for at least 1 year after recovery (26).

As described previously in our study, the surface markers OX40, CD40L, CD69, and CD25 were used to evaluate T cell activation. Selecting the most suitable activation marker for T lymphocytes depends on the specific context and objectives of the research or clinical application. It is important to note that the kinetics of activation markers differ, with peak expression occurring at different times during *in vitro* stimulation, depending on the marker and the nature of the stimulation protocol. For example, markers such as CD25 (IL-2 receptor alpha chain) and CD69 (an early T-cell activation marker) can peak within hours (2–24 hours) after stimulation (27, 28). CD154 (CD40 ligand) expression on T cells peaks around 6 to 24 hours, and is transient unless maintained by ongoing stimulatory signals (29). In contrast, cytokine production (e.g., IFN-γ, IL-2), typically peak later, generally between 24 to 72 hours after stimulation (30). The exact timing of peak expression can vary depending on factors like the type of T cell, the strength and duration of the stimulation, and the experimental conditions. In this context, adding a positive control is crucial to confirm that the stimulation has been effective, and it is especially important when there is uncertainty about the individual’s immunological status. Since CD69 was the only marker that consistently showed an increase after polyclonal stimulation, it indicates that CD69 is a reliable marker to assess T cell reactivity.

The AIM assay not only determines the activation status of CD4 and CD8 T cells but also enables the classification of T lymphocytes into functionally distinct populations. Whether TCM cells express more activation markers than TEM cells depends on the specific context of the immune response, the nature of the antigen exposure, and the tissue microenvironment (31). Typically, CD69 is highly expressed in effector T cells (32). However, when analyzing the total cohort of patients with CD4 reactivity, we found that the CM population was the most significantly increased. While TCM cells are not typically considered effector cells, they play a crucial role in rapidly generating effector cells upon antigen re-exposure. These findings provide a deeper analysis compared to other techniques and could potentially aid in better characterizing patients who are negative for CMV reactivity.

In our report, two patients with CD8+ T cell reactivity did not exhibit CD4+ reactivity. Conversely, one patient showed CD4+ reactivity but was CD8 negative. These findings may have clinical implications for patient management. Historically, CD8+ T cells have been considered the primary effectors in controlling viral infections and generating long-term immunity against CMV (33). However, the coordinated action of both CD4+ and CD8+ lymphocytes is essential for effective immune control. A deficiency in CMV-specific CD4+ T cells has been associated with an inability to control CMV infection (34–36). Furthermore, a reduction in CMV-specific CD4+ T cells, as assessed by flow cytometry, is a stronger predictor of CMV-related events in transplant patients than the reduction in CD8+ T cells (36). CD4+ T cells are also crucial in protecting patients from relapse and eliminating viremia. Given that both T cell subsets play vital roles in the immune response against CMV, these findings underscore the importance of testing both CD4+ and CD8+ T cell reactivity for a comprehensive and accurate interpretation (37).

Recently, Fernández-Ruiz et al., described the discriminative capacity to predict immune protection against clinically relevant CMV infection among intermediate-risk KT recipients was comparable for ICS and QTF-CMV. The authors also suggested a selected ICS threshold may provide better specificity than the interpretative cut-off values currently recommended for QTF-CMV (38).

In our comparative study, both ELISA assays demonstrated high sensitivity and specificity compared to the other techniques. The consistency test results further showed that the agreement between AIM (cut-off 0.2) and QF ELISA was perfect, and between AIM (cut-off 0.2) and ELISA was almost perfect. However, it is important to note that these results can vary depending on the patient’s immunological status. For example, in our study, three high-risk patients (D+/R-) tested negative on all four assays. This outcome does not indicate a lack of sensitivity or specificity of the tests but rather reflects a limited ability of these patients to mount an effective immune response to the virus. Additionally, when a patient does not respond to the positive control, the interpretation of the results becomes ambiguous, which may indicate global immunosuppression. While this did not affect our study, it highlights the potential for invalid results under such circumstances.

In clinical practice using the AIM assay, results that fall between 20% and 100% of the control were considered “borderline,” with patients showing similar clinical profiles to those who exhibited no reactivity. Although some studies using QF CMV have predicted responses based on the provided cut-offs (33), recent research suggests that these cut-off values should be validated more extensively (37, 39). The adequacy of these cut-offs in predicting a true immune response against CMV remains unclear and warrants further investigation.

The ELISA is commonly used in clinical diagnostics due to its low cost, simplicity, relatively high sensitivity, specificity and the ability to automate the test for quick results. However, a limitation of this in- house assay is the need to establish a cut-off based on the study population, which can be particularly challenging in transplant populations. Also, in-house testing could lack standardization, leading to less reliable results.

While ELISPOT and ICS are also sensitive assays that can be implemented in routine clinical practice, they were not included in our study, which represents a limitation of the research.

Furthermore, the choice of amino acid sequence used for test evaluation can directly impact the efficiency and sensitivity of *in vitro* tests designed to detect human cytomegalovirus (hCMV)-specific T cells (37). In this study, the TCPA, AIM, and ELISA assays were all stimulated using peptide pools derived from the pp65 protein, targeting both CD4+ and CD8+ T cells. In contrast, the QF CMV ELISA incorporates peptide pools from multiple viral proteins, including IE-1, IE-2, pp65, pp50, and gB, which specifically target CD8+ T lymphocytes. Additionally, the QF CMV ELISA’s peptide pool covers more than 98% of human haplotypes. These differences are important as they have direct implications for test sensitivity. In cases where patients test negative on the initial analysis, reviewing their HLA type or considering alternative methods may be necessary.

Moreover, immediate-early antigens, such as IE-1 and IE-2, are believed to play a crucial role in the early stages of CMV infection, while tegument-derived antigens, like pp65, become more relevant in later stages, particularly post-transplantation. Therefore, monitoring hCMV-specific T-cell responses should ideally involve a range of viral proteins to capture this diversity. This consideration has direct implications for sensitivity and represents a limitation of our study, as only pp65-derived peptide pools were used to stimulate the assays in our analysis (AIM, TCPA, and ELISA).

In a comparative cost analysis conducted at our center, the four assays under evaluation had similar costs. Thus, the selection of a test should depend more on the center’s experience with each method, access to flow cytometry platforms, and the established sensitivity and specificity with validated cut-off values.

Regarding regulatory aspects in Europe, the QF CMV ELISA is an IVD (*in vitro* diagnostic) device and is CE-marked, while the other assays (AIM, TCPA, and ELISA) are eligible for accreditation under the ISO 15189 quality system.

Considering factors such as feasibility for daily clinical use, sensitivity, specificity, and cost-effectiveness, our proposed algorithm for CMV monitoring is as follows: Initially, we test patients with CMV QF ELISA after confirming that their HLA type matches the Qiagen peptide pool (>98% coverage). For patients who are indeterminate or whose HLA types do not match the Qiagen peptides, we conduct AIM testing using both the IE-1 and pp65 proteins. In AIM, we consider results between 20% and 100% of the control as “borderline,” and we emphasize the need for validating the cut-offs used in the techniques applied. According to our clinical protocol, a positive result from any of these assays indicates stopping prophylactic antiviral treatment and initiating preemptive therapy based on the patient’s immune status.

## Data Availability

The original contributions presented in the study are included in the article/**Supplementary Material**. Further inquiries can be directed to the corresponding author.

## Supplementary material

Supplementary Figure 1 Gating strategy. After debris exclusion lymphocytes are gated based on size and density, the CD3+ population is selected from the lymphocyte population. Within the CD3+ population, CD8+ and CD4+ cells are analyzed. T cell differentiation is then analyzed from the CD4+ and CD8+ populations based on CCR7 and CD45RA expression: naïve (CCR7^+^ CD45RA^+^), CM (CCR7^−^ CD45RA), EM (CCR7^−^ CD45RA^-^) and EMRA (CCR7^−^ CD45RA) T-cell.

Supplementary Figure 2 Fluorescence Minus One controls (FMOs) to assess the background signal for the CD69 CD25, OX40, and CD40 Ligand (L) expression markers.

Supplementary Table 1 Type of test and number of samples classified by serostatus.

Supplementary Table 2 TCPA results.

Supplementary Table 3 AIM results, cut off >20% CN.

Supplementary Table 4 AIM, cut off >100% CN.

Supplementary Table 5 ELISA results.

Supplementary Table 6 QF CMV ELISA results.

Supplementary material 1 Consistency analysis of TCPA, AIM, ELISA and QF ELISA. Cohen’s kappa values were calculated using ChatGPT (GPT-4, developed by OpenAI).
