# Supplementary material for: Comparison of four different assays to evaluate cellular-mediated immunity against cytomegalovirus in solid organ transplantation
Source: Front Immunol. 2025 May 16;16:1567253. doi: 10.3389/fimmu.2025.1567253 (PMC12122514; doi:10.3389/fimmu.2025.1567253)
Supplement: Supplementary Figure 1 — Gating strategy. After debris exclusion lymphocytes are gated based on size and density, the CD3+ population is selected from the lymphocyte population. Within the CD3+ population, CD8+ and CD4+ cells are analyzed. T cell differentiation is then analyzed from the CD4+ and CD8+ populations based on CCR7 and CD45RA expression: naïve (CCR7+ CD45RA+), CM (CCR7− CD45RA), EM (CCR7− CD45RA-) and EMRA (CCR7− CD45RA) T-cell. [file Presentation1.zip › Supplementary figures, tables and material/Supplementary material 1. Cohen's kappa values.docx]

**Contingency Table TCPA vs AIM 0.2**

|  | **TCPA Positive** | **TCPA Negative** | **Total** |
| --- | --- | --- | --- |
| **AIM Positive** | **9** | **5** | **14** |
| **AIM Negative** | **0** | **11** | **11** |
| **Total** | **9** | **16** | **25** |

Steps to calculate Cohen's Kappa:

1. Observed Agreement (Po):

Po=9+1125P_o = \frac{9 + 11}{25}

1. Expected Agreement (Pe):

Pe=(9+5)(9+0)252+(5+11)(0+11)252P_e = \frac{(9+5)(9+0)}{25^2} + \frac{(5+11)(0+11)}{25^2}

1. Kappa Formula:

κ=Po−Pe1−Pe\kappa = \frac{P_o - P_e}{1 - P_e}

The results for the comparison between AIM cut off 0.2 and TCPA are as follows:

1. Observed Agreement (Po): 0.800 (80.0%)
2. Expected Agreement (Pe): 0.483 (48.3%)
3. Cohen's Kappa (κ): 0.613 (61.3%)

This indicates a substantial level of agreement between the two measures.

**Contingency Table TCPA vs AIM 2**

|  | **TCPA Positive** | **TCPA Negative** | **Total** |
| --- | --- | --- | --- |
| **AIM Positive** | **6** | **0** | **6** |
| **AIM Negative** | **3** | **16** | **19** |
| **Total** | **9** | **16** | **25** |

Steps to calculate Cohen's Kappa:

1. Observed Agreement (Po):

Po=6+1625P_o = \frac{6 + 16}{25}

1. Expected Agreement (Pe):

Pe=(6+0)(6+3)252+(0+16)(3+16)252P_e = \frac{(6+0)(6+3)}{25^2} + \frac{(0+16)(3+16)}{25^2}

1. Kappa Formula:

κ=Po−Pe1−Pe\kappa = \frac{P_o - P_e}{1 - P_e}

The results for the comparison between AIM cut off 2 and TCPA are as follows:

1. Observed Agreement (Po): 0.880 (88.0%)
2. Expected Agreement (Pe): 0.573 (57.3%)
3. Cohen's Kappa (κ): 0.719 (71.9%)

This indicates a substantial to almost perfect level of agreement between the two measures.

**Contingency Table ELISA vs AIM 0.2**

|  | **ELISA Positive** | **ELISA Negative** | **Total** |
| --- | --- | --- | --- |
| **AIM Positive** | **6** | **0** | **6** |
| **AIM Negative** | **1** | **5** | **6** |
| **Total** | **7** | **5** | **12** |

Steps to calculate Cohen's Kappa:

1. Observed Agreement (Po):

Po=6+512P_o = \frac{6 + 5}{12}

1. Expected Agreement (Pe):

Pe=(6+0)(6+1)122+(0+5)(1+5)122P_e = \frac{(6+0)(6+1)}{12^2} + \frac{(0+5)(1+5)}{12^2}

1. Kappa Formula:

κ=Po−Pe1−Pe\kappa = \frac{P_o - P_e}{1 - P_e}

The results for the comparison between AIM and ELISA are as follows:

1. Observed Agreement (Po): 0.917 (91.7%)
2. Expected Agreement (Pe): 0.500 (50.0%)
3. Cohen's Kappa (κ): 0.833 (83.3%)

This indicates a very strong level of agreement between the two measures.

**Contingency Table ELISA vs AIM 2**

|  | **ELISA Positive** | **ELISA Negative** | **Total** |
| --- | --- | --- | --- |
| **AIM Positive** | 3 | 0 | 3 |
| **AIM Negative** | 4 | 5 | 9 |
| **Total** | 7 | 5 | 12 |

Steps to Calculate Cohen's Kappa:

1. Observed Agreement (Po):

Po=3+512P_o = \frac{3 + 5}{12}

1. Expected Agreement (Pe):

Pe=(3+0)(3+4)122+(4+5)(0+5)122P_e = \frac{(3+0)(3+4)}{12^2} + \frac{(4+5)(0+5)}{12^2}

1. Kappa Formula:

κ=Po−Pe1−Pe\kappa = \frac{P_o - P_e}{1 - P_e}

The results for the comparison between AIM cut off 2 and ELISA are as follows:

1. Observed Agreement (Po): 0.667 (66.7%)
2. Expected Agreement (Pe): 0.458 (45.8%)
3. Cohen's Kappa (κ): 0.385 (38.5%)

This indicates a fair level of agreement between the two measures.

**Contingency Table ELISA vs TCPA**

|  | **ELISA Positive** | **ELISA Negative** | **Total** |
| --- | --- | --- | --- |
| **TCPA Positive** | 12 | 0 | 12 |
| **TCPA Negative** | 5 | 3 | 8 |
| **Total** | 17 | 3 | 20 |

Steps to Calculate Cohen's Kappa:

1. Observed Agreement (Po):

Po=12+320P_o = \frac{12 + 3}{20}

1. Expected Agreement (Pe):

Pe=(12+0)(12+5)202+(5+3)(0+3)202P_e = \frac{(12+0)(12+5)}{20^2} + \frac{(5+3)(0+3)}{20^2}

1. Kappa Formula:

κ=Po−Pe1−Pe\kappa = \frac{P_o - P_e}{1 - P_e}

Here are the results for Cohen's Kappa calculation between TCPA and ELISA:

1. Observed Agreement (Po): 0.75
2. Expected Agreement (Pe): 0.57
3. Cohen's Kappa (κ): 0.419

This indicates a moderate level of agreement.

**Contingency Table ELISA vs QF ELISA**

|  | **QF ELISA Positive** | **QF ELISA Negative** | **Total** |
| --- | --- | --- | --- |
| **ELISA Positive** | 14 | 3 | 17 |
| **ELISA Negative** | 0 | 4 | 4 |
| **Total** | 14 | 7 | 21 |

Steps to Calculate Cohen's Kappa:

1. Observed Agreement (Po):

Po=14+421P_o = \frac{14 + 4}{21}

1. Expected Agreement (Pe):

Pe=(14+3)(14+0)212+(3+4)(0+7)212P_e = \frac{(14+3)(14+0)}{21^2} + \frac{(3+4)(0+7)}{21^2}

1. Kappa Formula:

κ=Po−Pe1−Pe\kappa = \frac{P_o - P_e}{1 - P_e}

The calculation of Cohen's Kappa index with the new dataset yields the following results:

- PoP_o (observed agreement proportion): 0.8570.857
- PeP_e (expected agreement proportion by chance): 0.6030.603
- Kappa: 0.6400.640

This indicates a moderate to substantial agreement between the QF ELISA and ELISA tests, according to the standard Cohen's Kappa interpretation scale.

**Contingency Table QF ELISA VS AIM 0.2**

|  | **QF ELISA Positive** | **QF ELISA Negative** | **Total** |
| --- | --- | --- | --- |
| **AIM cut off 0.2 Positive** | 24 | 0 | 24 |
| **AIM cut off 0.2 Negative** | 0 | 13 | 13 |
| **Total** | 24 | 13 | 37 |

Steps to Calculate Cohen's Kappa:

1. Observed Agreement (Po):

Po=24+1337P_o = \frac{24 + 13}{37}

1. Expected Agreement (Pe):

Pe=(24+0)(24+13)372+(0+13)(13+13)372P_e = \frac{(24+0)(24+13)}{37^2} + \frac{(0+13)(13+13)}{37^2}

1. Kappa Formula:

κ=Po−Pe1−Pe\kappa = \frac{P_o - P_e}{1 - P_e}

Here are the results for Cohen's Kappa calculation:

1. Observed Agreement (Po): 1.0
2. Expected Agreement (Pe): 0.5442
3. Cohen's Kappa (κ): 1.0

This indicates perfect agreement between AIM cut off 0.2 and QF ELISA, as Kappa equals 1.

**Contingency Table QF ELISA VS AIM 2**

|  | QF ELISA Positive | QF ELISA Negative | Total |
| --- | --- | --- | --- |
| AIM cut off 2 Positive | 9 | 0 | 9 |
| AIM cut off 2 Negative | 15 | 13 | 28 |
| Total | 24 | 13 | 37 |

Steps to Calculate Cohen's Kappa:

1. Observed Agreement (Po):

Po=9+1337P_o = \frac{9 + 13}{37}

1. Expected Agreement (Pe):

Pe=(9+0)(24)+(15+13)(13)372P_e = \frac{(9+0)(24) + (15+13)(13)}{37^2}

1. Kappa Formula:

κ=Po−Pe1−Pe\kappa = \frac{P_o - P_e}{1 - P_e}

Here are the results for Cohen's Kappa calculation between AIM cut off 2 and QF ELISA:

1. Observed Agreement (Po): 0.5946
2. Expected Agreement (Pe): 0.4237
3. Cohen's Kappa (κ): 0.2966

This indicates a fair level of agreement.

**Contingency Table QF ELISA VS TCPA**

|  | **QF ELISA Positive** | **QF ELISA Negative** | **Total** |
| --- | --- | --- | --- |
| **TCPA Positive** | 18 | 0 | 18 |
| **TCPA Negative** | 7 | 12 | 19 |
| **Total** | 25 | 12 | 37 |

Steps to Calculate Cohen's Kappa:

1. Observed Agreement (Po):

Po=18+1237P_o = \frac{18 + 12}{37}

1. Expected Agreement (Pe):

Pe=(18+7)(25)+(0+12)(12)372P_e = \frac{(18+7)(25) + (0+12)(12)}{37^2}

1. Kappa Formula:

κ=Po−Pe1−Pe\kappa = \frac{P_o - P_e}{1 - P_e}

Here are the results for Cohen's Kappa calculation between TCPA and QF ELISA:

1. Observed Agreement (Po): 0.811
2. Expected Agreement (Pe): 0.495
3. Cohen's Kappa (κ): 0.625

This indicates a substantial level of agreement.
