# Supplementary material for: Comparison of four different assays to evaluate cellular-mediated immunity against cytomegalovirus in solid organ transplantation
Source: Front Immunol. 2025 May 16;16:1567253. doi: 10.3389/fimmu.2025.1567253 (PMC12122514; doi:10.3389/fimmu.2025.1567253)
Supplement: Supplementary Figure 1 — Gating strategy. After debris exclusion lymphocytes are gated based on size and density, the CD3+ population is selected from the lymphocyte population. Within the CD3+ population, CD8+ and CD4+ cells are analyzed. T cell differentiation is then analyzed from the CD4+ and CD8+ populations based on CCR7 and CD45RA expression: naïve (CCR7+ CD45RA+), CM (CCR7− CD45RA), EM (CCR7− CD45RA-) and EMRA (CCR7− CD45RA) T-cell. [file Presentation1.zip › Supplementary figures, tables and material/Supplementary material 1. Cohen's kappa values.pdf]

Contingency Table TCPA vs AIM 0.2

	TCPA Positive	TCPA Negative	Total
**AIM Positive**	**9**	**5**	**14**
**AIM Negative**	**0**	**11**	**11**
**Total**	**9**	**16**	**25**

Steps to calculate Cohen's Kappa:

1. Observed Agreement (Po):

Po=9+1125P_o = \frac{9 + 11}{25}

2. Expected Agreement (Pe):

Pe=(9+5)(9+0)252+(5+11)(0+11)252P_e = \frac{(9+5)(9+0)}{25^2} + \frac{(5+11)(0+11)}{25^2}

3. Kappa Formula:

κ=Po−Pe1−Pe\kappa = \frac{P_o - P_e}{1 - P_e}

The results for the comparison between AIM cut off 0.2 and TCPA are as follows:

1. Observed Agreement (Po): 0.800 (80.0%)
2. Expected Agreement (Pe): 0.483 (48.3%)
3. Cohen's Kappa (κ): 0.613 (61.3%)

This indicates a substantial level of agreement between the two measures.

Contingency Table TCPA vs AIM 2

	TCPA Positive	TCPA Negative	Total
**AIM Positive**	**6**	**0**	**6**
**AIM Negative**	**3**	**16**	**19**
**Total**	**9**	**16**	**25**

Steps to calculate Cohen's Kappa:

1. Observed Agreement (Po):

Po=6+1625P_o = \frac{6 + 16}{25}

2. Expected Agreement (Pe):

Pe=(6+0)(6+3)252+(0+16)(3+16)252P_e = \frac{(6+0)(6+3)}{25^2} + \frac{(0+16)(3+16)}{25^2}

3. Kappa Formula:

κ=Po−Pe1−Pe\kappa = \frac{P_o - P_e}{1 - P_e}

The results for the comparison between AIM cut off 2 and TCPA are as follows:

1. Observed Agreement (Po): 0.880 (88.0%)
2. Expected Agreement (Pe): 0.573 (57.3%)
3. Cohen's Kappa (κ): 0.719 (71.9%)

This indicates a substantial to almost perfect level of agreement between the two measures.

Contingency Table ELISA vs AIM 0.2

	**ELISA Positive**	**ELISA Negative**	**Total**
**AIM Positive**	**6**	**0**	**6**
**AIM Negative**	**1**	**5**	**6**
**Total**	**7**	**5**	**12**

Steps to calculate Cohen's Kappa:

1. Observed Agreement (Po):

Po=6+512P_o = \frac{6 + 5}{12}

2. Expected Agreement (Pe):

Pe=(6+0)(6+1)122+(0+5)(1+5)122P_e = \frac{(6+0)(6+1)}{12^2} + \frac{(0+5)(1+5)}{12^2}

3. Kappa Formula:

κ=Po−Pe1−Pe\kappa = \frac{P_o - P_e}{1 - P_e}

The results for the comparison between AIM and ELISA are as follows:

1. Observed Agreement (Po): 0.917 (91.7%)
2. Expected Agreement (Pe): 0.500 (50.0%)
3. Cohen's Kappa (κ): 0.833 (83.3%)

This indicates a very strong level of agreement between the two measures.

Contingency Table ELISA vs AIM 2

	**ELISA Positive**	**ELISA Negative**	**Total**
**AIM Positive**	3	0	3
**AIM Negative**	4	5	9
**Total**	7	5	12

Steps to Calculate Cohen's Kappa:

1. Observed Agreement (Po):

   Po=3+512P_o = \frac{3 + 5}{12}

2. Expected Agreement (Pe):

   Pe=(3+0)(3+4)122+(4+5)(0+5)122P_e = \frac{(3+0)(3+4)}{12^2} + \frac{(4+5)(0+5)}{12^2}

3. Kappa Formula:

   κ=Po−Pe1−Pe\kappa = \frac{P_o - P_e}{1 - P_e}

The results for the comparison between AIM cut off 2 and ELISA are as follows:

1. Observed Agreement (Po): 0.667 (66.7%)
2. Expected Agreement (Pe): 0.458 (45.8%)
3. Cohen's Kappa (κ): 0.385 (38.5%)

This indicates a fair level of agreement between the two measures.

Contingency Table ELISA vs TCPA

	ELISA Positive	ELISA Negative	Total
**TCPA Positive**	12	0	12
**TCPA Negative**	5	3	8
**Total**	17	3	20

Steps to Calculate Cohen's Kappa:

1. Observed Agreement (Po):

   Po=12+320P_o = \frac{12 + 3}{20}

2. Expected Agreement (Pe):

   Pe=(12+0)(12+5)202+(5+3)(0+3)202P_e = \frac{(12+0)(12+5)}{20^2} + \frac{(5+3)(0+3)}{20^2}

3. Kappa Formula:

   κ=Po−Pe1−Pe\kappa = \frac{P_o - P_e}{1 - P_e}

Here are the results for Cohen's Kappa calculation between TCPA and ELISA:

1. Observed Agreement (Po): 0.75
2. Expected Agreement (Pe): 0.57
3. Cohen's Kappa (κ): 0.419

This indicates a moderate level of agreement.

Contingency Table ELISA vs QF ELISA

	QF ELISA Positive	QF ELISA Negative	Total
**ELISA Positive**	14	3	17
**ELISA Negative**	0	4	4
**Total**	14	7	21

Steps to Calculate Cohen's Kappa:

1. Observed Agreement (Po):

Po=14+421P_o = \frac{14 + 4}{21}

2. Expected Agreement (Pe):

Pe=(14+3)(14+0)212+(3+4)(0+7)212P_e = \frac{(14+3)(14+0)}{21^2} + \frac{(3+4)(0+7)}{21^2}

3. Kappa Formula:

κ=Po−Pe1−Pe\kappa = \frac{P_o - P_e}{1 - P_e}

The calculation of Cohen's Kappa index with the new dataset yields the following results:

- PoP_o (observed agreement proportion): 0.8570.857
- PeP_e (expected agreement proportion by chance): 0.6030.603
- Kappa: 0.6400.640

This indicates a moderate to substantial agreement between the QF ELISA and ELISA tests, according to the standard Cohen's Kappa interpretation scale.

Contingency Table QF ELISA VS AIM 0.2

	QF ELISA Positive	QF ELISA Negative	Total
**AIM cut off 0.2 Positive**	24	0	24
**AIM cut off 0.2 Negative**	0	13	13
**Total**	24	13	37

Steps to Calculate Cohen's Kappa:

1. Observed Agreement (Po):

   Po=24+1337P_o = \frac{24 + 13}{37}

2. Expected Agreement (Pe):

   Pe=(24+0)(24+13)372+(0+13)(13+13)372P_e = \frac{(24+0)(24+13)}{37^2} + \frac{(0+13)(13+13)}{37^2}

3. Kappa Formula:

   κ=Po−Pe1−Pe\kappa = \frac{P_o - P_e}{1 - P_e}

Here are the results for Cohen's Kappa calculation:

1. Observed Agreement (Po): 1.0
2. Expected Agreement (Pe): 0.5442
3. Cohen's Kappa (κ): 1.0

This indicates perfect agreement between AIM cut off 0.2 and QF ELISA, as Kappa equals 1.

Contingency Table QF ELISA VS AIM 2

	QF ELISA Positive	QF ELISA Negative	Total
AIM cut off 2 Positive	9	0	9
AIM cut off 2 Negative	15	13	28
Total	24	13	37

Steps to Calculate Cohen's Kappa:

1. Observed Agreement (Po):

   Po=9+1337P_o = \frac{9 + 13}{37}

2. Expected Agreement (Pe):

   Pe=(9+0)(24)+(15+13)(13)372P_e = \frac{(9+0)(24) + (15+13)(13)}{37^2}

3. Kappa Formula:

   κ=Po−Pe1−Pe\kappa = \frac{P_o - P_e}{1 - P_e}

Here are the results for Cohen's Kappa calculation between AIM cut off 2 and QF ELISA:

1. Observed Agreement (Po): 0.5946
2. Expected Agreement (Pe): 0.4237
3. Cohen's Kappa (κ): 0.2966

This indicates a fair level of agreement.

Contingency Table QF ELISA VS TCPA

	**QF ELISA Positive**	**QF ELISA Negative**	**Total**
**TCPA Positive**	18	0	18
**TCPA Negative**	7	12	19
**Total**	25	12	37

Steps to Calculate Cohen's Kappa:

1. Observed Agreement (Po):

   Po=18+1237P_o = \frac{18 + 12}{37}

2. Expected Agreement (Pe):

   Pe=(18+7)(25)+(0+12)(12)372P_e = \frac{(18+7)(25) + (0+12)(12)}{37^2}

3. Kappa Formula:

   κ=Po−Pe1−Pe\kappa = \frac{P_o - P_e}{1 - P_e}

Here are the results for Cohen's Kappa calculation between TCPA and QF ELISA:

1. Observed Agreement (Po): 0.811
2. Expected Agreement (Pe): 0.495
3. Cohen's Kappa (κ): 0.625

This indicates a substantial level of agreement.
