# Supplementary figures and images for: Comparison of four different assays to evaluate cellular-mediated immunity against cytomegalovirus in solid organ transplantation
Source: Front Immunol. 2025 May 16;16:1567253. doi: 10.3389/fimmu.2025.1567253 (PMC12122514; doi:10.3389/fimmu.2025.1567253)

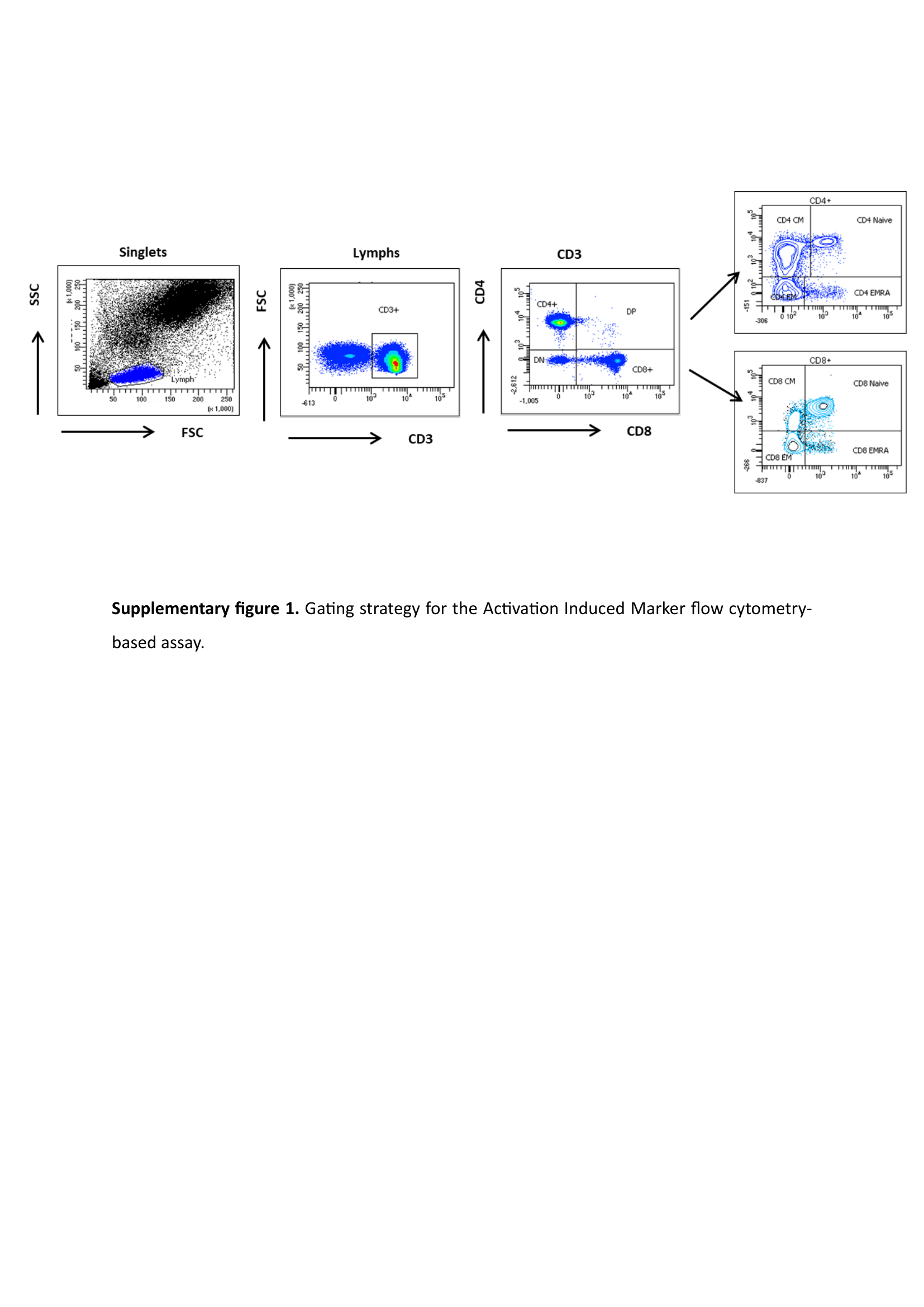

Supplement: Supplementary Figure 1 — Gating strategy. After debris exclusion lymphocytes are gated based on size and density, the CD3+ population is selected from the lymphocyte population. Within the CD3+ population, CD8+ and CD4+ cells are analyzed. T cell differentiation is then analyzed from the CD4+ and CD8+ populations based on CCR7 and CD45RA expression: naïve (CCR7+ CD45RA+), CM (CCR7− CD45RA), EM (CCR7− CD45RA-) and EMRA (CCR7− CD45RA) T-cell. [file Presentation1.zip › Supplementary figures, tables and material/Supplementary figure 1. Gating strategy..tiff]

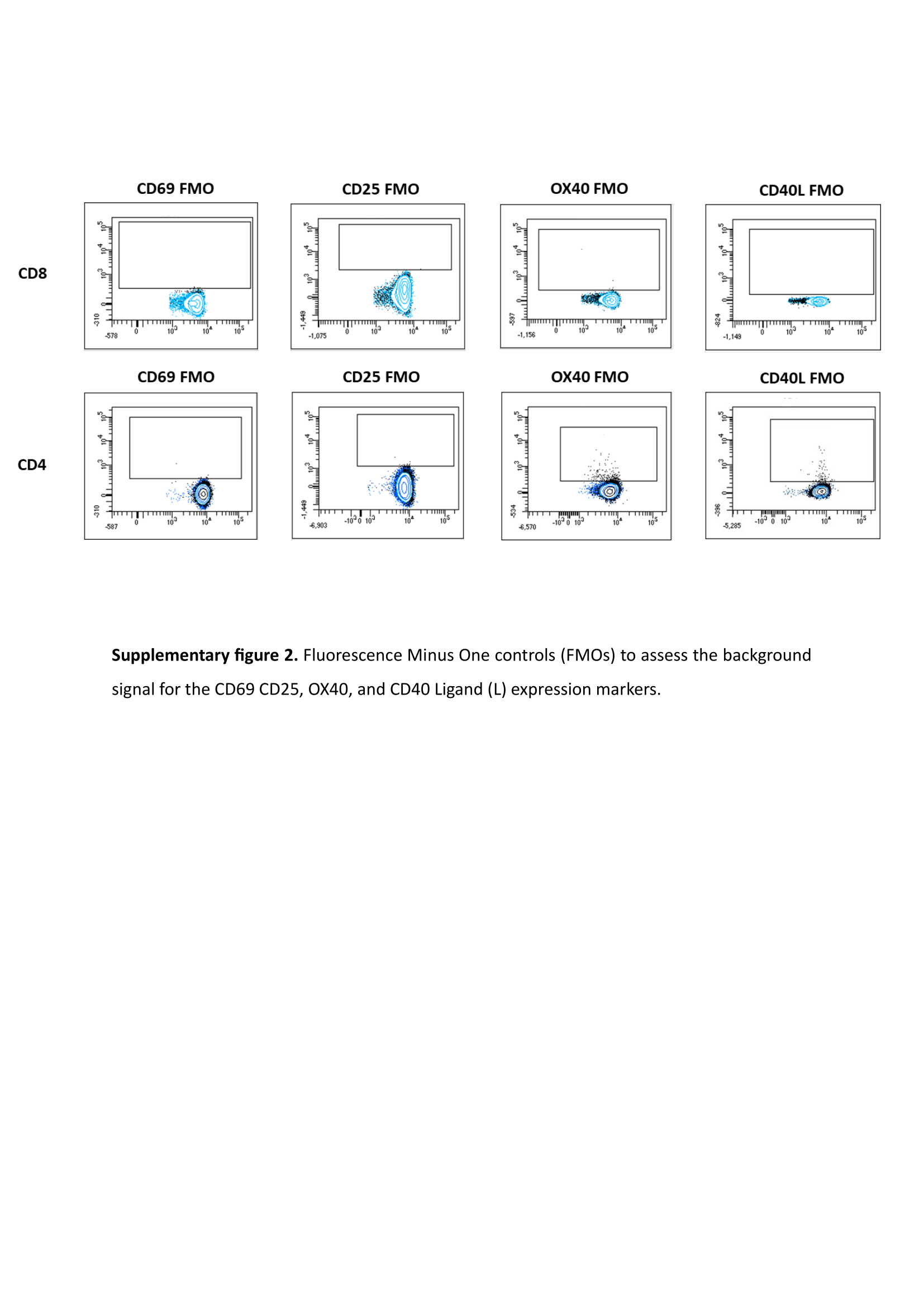

Supplement: Supplementary Figure 1 — Gating strategy. After debris exclusion lymphocytes are gated based on size and density, the CD3+ population is selected from the lymphocyte population. Within the CD3+ population, CD8+ and CD4+ cells are analyzed. T cell differentiation is then analyzed from the CD4+ and CD8+ populations based on CCR7 and CD45RA expression: naïve (CCR7+ CD45RA+), CM (CCR7− CD45RA), EM (CCR7− CD45RA-) and EMRA (CCR7− CD45RA) T-cell. [file Presentation1.zip › Supplementary figures, tables and material/Supplementary figure 2..tiff]
